# Prospecting of the Antioxidant Activity from Extracts Obtained from Chañar (*Geoffroea decorticans*) Seeds Evaluated In Vitro and In Vivo Using the *Tenebrio molitor* Model

**DOI:** 10.3390/nu16172813

**Published:** 2024-08-23

**Authors:** Ariana Pereira Silva, Maria Lucia da Silva Cordeiro, Verônica Giuliani de Queiroz Aquino-Martins, Luciana Fentanes de Moura Melo, Weslley de Souza Paiva, Georggia Fatima da Silva Naliato, Raquel Cordeiro Theodoro, Carlos Henrique Salvino Gadelha Meneses, Hugo Alexandre Oliveira Rocha, Katia Castanho Scortecci

**Affiliations:** 1 Laboratório de Transformação de Plantas e Análise em Microscopia (LTPAM), Departamento de Biologia Celular e Genética, Universidade Federal do Rio Grande do Norte (UFRN), Natal 59078-970, RN, Brazil; arianapereirauf@gmail.com (A.P.S.); mlscordeiro04@gmail.com (M.L.d.S.C.); verinhaquino@hotmail.com (V.G.d.Q.A.-M.); lucianafentanes@gmail.com (L.F.d.M.M.); 2 Programa de Pós-Graduação em Bioquímica e Biologia Molecular, Centro de Biociências, Universidade Federal do Rio Grande do Norte (UFRN), Natal 59078-970, RN, Brazil; georggianaliato@gmail.com (G.F.d.S.N.); raquel.theodoro@ufrn.br (R.C.T.); hugo.rocha@ufrn.br (H.A.O.R.); 3 Laboratório de Biotecnologia de Polímeros Naturais (BIOPOL), Departamento de Bioquímica, Universidade Federal do Rio Grande do Norte (UFRN), Natal 59078-970, RN, Brazil; wdspaiva@gmail.com; 4 Instituto de Medicina Tropical, Universidade Federal do Rio Grande do Norte (UFRN), Natal 59077-080, RN, Brazil; 5 Laboratório de Biotecnologia Vegetal (LBV), Departamento de Biologia, Centro de Ciências Biológicas e da Saúde, Universidade Estadual da Paraiba (UEPB), Campina Grande 58429-500, PB, Brazil; carlos.meneses@gsuite.uepb.edu.br

**Keywords:** ethanolic extract, aqueous extract, phenolic compounds, oxidative stress, copper sulphate, *Tenebrio molitor* model, antioxidant protection activity

## Abstract

*Geoffroea decorticans,* commonly known as Chañar, is a native Chilean plant widely used in folk medicine for its expectorant, pain relief, and antinociceptive properties. This study explored the antioxidant, cytotoxic, and protective effects of its ethanolic (EE) and aqueous (EA) seed extracts against oxidative stress induced by copper sulfate, using both in vitro and in vivo approaches. Phytochemical analyses revealed the presence of phenolic compounds and flavonoids in the extracts. High-Performance Liquid Chromatography (HPLC) coupled with Gas Chromatography-Mass Spectrometry/Mass Spectrometry (GC-MS/MS) identified significant components such as phytol, alpha-tocopherol, vitexin, and rutin, with the EE being particularly rich in phytol and vitexin. Antioxidant assays—measuring the total antioxidant capacity (TAC), reducing power, DPPH radical scavenging, and copper and iron chelation—confirmed their potent antioxidant capabilities. Both extracts were non-cytotoxic and provided protection against CuSO_4_-induced oxidative stress in the 3T3 cell line. Additionally, the use of *Tenebrio molitor* as an invertebrate model underscored the extracts’ antioxidant and protective potentials, especially that of the EE. In conclusion, this study highlights the significant antioxidant and protective properties of Chañar seed extracts, particularly the ethanolic extract, in both in vitro and in vivo models.

## 1. Introduction

Reactive oxygen species (ROS) and reactive nitrogen species (RNS) are generated by all aerobic cells, either from endogenous metabolic processes or exogenous sources. When produced in appropriate amounts, these species play beneficial roles such as generating energy via the electron transport chain, participating in defense mechanisms during infections, and acting as signaling molecules, among other functions. In a physiologically normal state, cells can tolerate certain levels of ROS and RNS to maintain redox homeostasis [1].

However, when there is an imbalance between the production and degradation of these free radicals, cellular damage can occur [2]. Excessive ROS and RNS can harm macromolecules such as DNA, proteins, carbohydrates, and lipids, causing oxidative stress. This oxidative stress is linked to various diseases, including cancer, cardiovascular diseases, diabetes mellitus, chronic inflammatory conditions (e.g., asthma, arthritis, rheumatic diseases, fibromyalgia, ADHD, and hypertension), Alzheimer’s disease, Parkinson’s disease, and aging [1,3,4,5,6].

To mitigate this redox imbalance, cells express various antioxidant enzymes, including catalase, superoxide dismutase, and additional enzymatic systems, to efficiently counteract the deleterious effects of excessive ROS and RNS. Additionally, some antioxidant molecules can be obtained through exogenous supplementation in the diet, such as vitamins C and E, phenolic acids, flavonoids, anthocyanins, and other plant-produced intermediate metabolites [3,7,8].

Historically, various plant parts—leaves, flowers, fruits, seeds, and bark—have been utilized for medicinal purposes. This age-old knowledge, transmitted across generations, plays a crucial role in the research and development of new pharmaceuticals [9,10]. These medicinal plants have shown effectiveness in mitigating numerous chronic conditions, notably cardiovascular diseases, diabetes mellitus, and neurodegenerative diseases such as Alzheimer’s and Parkinson’s. They also serve a preventive role against cancer [11]. The efficacy of these plants is attributed to their rich biomolecular composition, which exhibits a range of biological effects, including antioxidant, anti-inflammatory, anticancer, antibacterial/antiviral, anti-osteoclastogenic, anti-diabetic, neuroprotective, and vasodilatory properties [12,13,14]. Consequently, ongoing research into identifying new bioactive molecules, particularly antioxidants such as phenolic compounds, is essential for enhancing therapeutic applications and maintaining cellular redox homeostasis [15,16,17,18,19,20].

The native tree of Chile, *Geoffroea decorticans* (Gill. ex Hook. & Arn.) Burkart (Fabaceae), commonly known as Chañar, is a deciduous and xerophytic species that can be found as either shrubs or trees. It grows most abundantly in the arid regions of northern Chile and Argentina, where the climate is similar to that of the Caatinga, a semi-arid biome that is exclusive to Brazil. In Brazil, within the Caatinga biome, *Geoffroea spinosa*, popularly known as Umari, Mari, or Marizeiro, a plant of the same genus as Chañar, is found. It is widely used as a food source and in folk medicine. Chañar is also extensively utilized by the indigenous people of the Atacama Desert. Various parts of this plant are well-adapted to the region’s limited water resources, offering valuable nutritional and medicinal benefits [21].

In folk medicine, the fruits of *G. decorticans* are widely used as an expectorant for treating bronchopulmonary disorders and for pain relief and antinociceptive properties [22]. Research also highlights their anticancer, anti-rheumatic, anti-inflammatory, and antifungal effects [23,24,25]. The seeds, commonly consumed after roasting, are favored for their almond-like taste and high oil content, contributing both flavor and nutritional benefits [26]. Moreover, the fruits are utilized in the preparation of syrups, known as arrope, and in flour-based products, which are rich in carbohydrates, proteins, potassium, fiber, and polyphenols [27]. Regarding its chemical composition, studies conducted by Maestri et al. [28] on two varieties of *G. decorticans* using the seeds showed that, on average, 48% of the dry matter consists of oil and 23% of protein; however, values for the total sugar content were not identified. Although the seeds of *G. decorticans* are a rich source of proteins and oils, such as oleic and linoleic acids [28], they are often discarded as waste after the fruit has been used [29]. Despite several previous studies reporting on the activities of the fruit, the phytochemical composition and antioxidant activity of *G. decorticans* seeds have received little attention and remain largely uncharacterized in terms of their potential.

Following the identification of the diverse traditional uses and nutritional contributions of *Geoffroea decorticans* seeds, this study explored in detail the phytochemical properties of the ethanol extract (EE) and aqueous extract (EA) derived from them. The antioxidant activities of these extracts were thoroughly assessed through biochemical in vitro assays and experiments with the 3T3 cell line. Furthermore, the antioxidant potential was also evaluated using the *Tenebrio molitor* model, a flour beetle from the family Tenebrionidae and Order Coleoptera. This particular model was selected due to its ease of handling, rapid growth, cost-effective reproduction, and suitably large larval body size, which supports inoculation and hemolymph collection. Employing this model adheres to the 3Rs principle—refinement, replacement, and reduction—in the bioethics of animal experimentation [30,31,32,33].

In this context, this study aimed to evaluate the antioxidant effects of the ethanolic and aqueous extracts of Chañar (*Geoffroea decorticans*) seeds both in vitro, using biochemical assays and a cell model (3T3 ATCC CCL-92), and in vivo. This study not only sought to reinforce the use of these seed extracts but also to provide scientific evidence of their antioxidant properties. This research opens the way for future studies and broadens the understanding of the potential applications of these seeds, whether in pharmacological, cosmetic, nutraceutical, or other fields.

## 2. Materials and Methods

### 2.1. Materials

Ethylenediaminetetraacetic acid (EDTA), gallic acid, ascorbic acid, quercetin, methionine, pirocatechol violet, riboflavin, and ammonium molybdate were obtained from Sigma-Aldrich Co. (St. Louis, MO, USA). Potassium ferricyanide, trichloroacetic acid, Folin–Ciocalteu reagent, and sulfuric acid were acquired from Merck (Darmstadt, Germany). 2,2-Diphenyl-1-picrylhydrazyl (DPPH) was sourced from Fluka (Seelze, Germany), and copper sulfate was obtained from Chemical Kinetics. Dulbecco’s Modified Eagle Medium (DMEM) and fetal bovine serum (FBS) were procured from CULTILAB (Campinas, SP, Brazil). Penicillin and streptomycin were sourced from Gibco (Fort Worth, TX, USA). All other reagents used were of analytical grade.

### 2.2. Plant Material

*Geoffroea decorticans* seeds were purchased online. Upon arrival at the laboratory, a portion of the seeds was allocated for extract preparation for the tests, while the remainder was stored in a dry, light-protected environment.

### 2.3. Extraction of Ethanolic and Aqueous Extracts from Geoffroea decorticans Seeds

Plant samples (seeds) were crushed/macerated to prepare extracts of varying polarity. One hundred grams of plant material was used per liter of solvent (1:10), following this order: hexane, chloroform, ethanol, methanol, and water [34]. An aqueous extract was prepared under identical conditions. For sequential extraction, the seed–solvent mixture was placed in an Erlenmeyer flask, set in a foam cooler with ice, sealed with tape, and shaken at 150 rpm for 24 h. After this period, the extract (liquid portion) was decanted and filtered through a Whatman No. 1 paper. Subsequently, the next solvent in the order of increasing polarity was added to the same plant material, culminating with water as the final solvent. This process resulted in six extracts: hexane (EH), chloroform (EC), ethanol (EE), methanol (EM), final water (EAF), and an additional aqueous extract (EA). After filtration, the extracts were dried using a Rotavapor (Tecnal, Sao Paulo, Brazil) at 40 °C. The resulting pasty extracts were dissolved in DMSO, constituting less than 1% of the final volume (Merck, Darmstadt, Germany), and then were lyophilized using a freeze dryer (Labconco FreeZone 4.5, SciQuip Ltda., Newtown, UK) to yield a powder. These powders were resuspended in water to achieve a final concentration of 100 mg/mL (stock solution) and stored in a freezer at −18 °C until needed.

### 2.4. Phytochemical Characterization

#### 2.4.1. Total Phenolic Compound Determination

The quantification of total phenolic compounds in the ethanolic and aqueous extracts was performed using the Folin–Ciocalteu colorimetric method, with gallic acid as a reference standard. A calibration curve for gallic acid was constructed using concentrations ranging from 0.1 mg/mL to 5 mg/mL (Supplementary Figure S1). The Folin–Ciocalteu reagent oxidizes the phenolic compounds in the sample, resulting in a color change that can be measured at 760 nm using a spectrophotometer (Epoch Biotek, Agilent Technologies, Santa Clara, CA, USA) [35].

#### 2.4.2. Quantification of Total Flavonoid Content

The concentration of flavonoids was determined using a modified aluminum chloride (AlCl_3_) method, as described by [36]. Briefly, 10 μL of the extracts (10 mg/mL) was added to each well of the plate, and the volume was brought up to 50 μL with distilled water. Subsequently, 160 μL of ethanol P.A. was added. This was followed by the addition of 20 μL of aluminum chloride solution (1.8%) and 20 μL of sodium acetate solution (8.2%) to the wells. The mixture was incubated for 40 min in the dark, after which the absorbance was measured at 415 nm using a spectrophotometer (Epoch Biotek). A standard curve was constructed with quercetin, ranging from 1.25 to 75 μg/mL. The results were expressed in milligrams of quercetin equivalents per gram of extract.

#### 2.4.3. Identification of Phenolic Compounds by HPLC Followed by GC-MS/SIM

The analysis of bioactive compounds was conducted using high-performance liquid chromatography (HPLC) followed by gas chromatography-mass spectrometry with selected ion monitoring (GC-MS/SIM). Initially, the samples were lyophilized and then dissolved in 1.5 mL of methanol for reverse-phase HPLC analysis using a Bondapak C18 column (3.9 mm × 300 mm, Waters Corp., Milford, MA, USA). The elution was carried out at a flow rate of 1.5 mL/min, with the mobile phases programmed as follows: from 0 to 5 min, 28% methanol in 1% aqueous acetic acid (isocratic); from 5 to 35 min, a linear gradient from 28% to 86% methanol; from 35 to 36 min, 86% to 100% methanol; and from 36 to 40 min, 100% methanol (isocratic). A total of 50 fractions of 1.5 mL each were collected and prepared for GC-MS analysis in SIM mode (GC system 6890N and selective mass detector 5973, Agilent Technologies, Santa Clara, CA, USA). For each type of substance detected, 1 μL of the sample was injected into a DB-1 capillary column (30 mm × 0.25 mm ID, 0.25 μm film thickness, J & W Scientific Co., Folsom, CA, USA). The GC oven temperature was programmed to start at 60 °C for 1 min, followed by an increase of 15 °C/min up to 200 °C, and then 5 °C/min to a final temperature of 285 °C. Helium was used as the carrier gas at a pressure of 30 kPa. The GC was directly coupled to a selective mass spectrometer, set with an interface and source temperature of 280 °C, an ionization voltage of 70 V, and a dwell time of 100 ms. The analysis was conducted in both full scan mode (initial test) and SIM mode, monitoring the nine main ions of the internal standards (phytol, vitexin, rutin, α-tocopherol, Amonafide, Caffeic acid, Isovitexin, Epifriedelanol, and Leucocyanidin) along with the detected compounds. Retention times were determined using hydrocarbon standards, and the quantification of substances was based on the peak ratios of non-deuterated compounds (extracted) to deuterated standards.

### 2.5. Evaluation of In Vitro Antioxidant Activity

Antioxidant compounds can be categorized into two main groups: primary (chain-breaking) antioxidants and secondary (preventive) antioxidants. Primary antioxidants primarily function by donating hydrogen atoms to free radicals, effectively neutralizing them. On the other hand, secondary antioxidants operate through multiple mechanisms, including scavenging reactive oxygen species, and metal chelation.

In this study, two types of *G. decorticans* seed extracts (aqueous and alcoholic) were evaluated, as they may contain different antioxidant compounds with varying mechanisms of action. To assess the in vitro antioxidant activity of these extracts, five distinct tests were conducted: Total Antioxidant Capacity (TAC), reducing power, 2,2-Diphenyl-1-picrylhydrazyl (DPPH) test, which measure the presence of primary antioxidants; as well as copper chelation and iron chelation tests, which evaluate the presence of secondary antioxidants. The concentrations of extract used in the tests were 50, 100, and 250 µg/mL.

#### 2.5.1. Total Antioxidant Capacity (TAC)

The antioxidant capacity of the plant extracts was evaluated using a colorimetric test, with results expressed in milligrams of ascorbic acid equivalents per gram of extract (mgE-AA/g Extract). The TAC aims to evaluate the total electron donating power of a compound. The application of a high temperature, acidic pH, and a strong oxidizing agent ensures that all molecules with electron donating potential will be evaluated in the assay, even if their tendency to donate electrons is weak [37].

This assay involves the reduction of Mo^6+^ to Mo^5+^ by active compounds in both the ethanolic and aqueous extracts, leading to the formation of a green phosphate/Mo^5+^ complex under acidic conditions [37]. For the procedure, tubes containing the extracts and a reagent solution—0.6 M sulfuric acid, 28 mM sodium phosphate, and 4 mM ammonium molybdate—were incubated at 95 °C for 90 min. Upon cooling to room temperature, the absorbance was measured at 695 nm using a spectrophotometer. The results were then compared to those of a negative control, which consisted of tubes containing all reagents except the plant extract.

#### 2.5.2. Reducing Power

Ethanolic and Aqueous Extracts were incubated with a phosphate buffer solution (0.2 M, pH 6.6) and 1% potassium ferricyanide at 50 °C for 20 min, with a final volume of 4 mL. The reaction was terminated by adding 10% trichloroacetic acid, followed by homogenization with distilled water and 0.1% ferric chloride. Finally, the absorbance was measured at 700 nm using a spectrophotometer (Epoch Biotek) [35,38].

#### 2.5.3. DPPH Radical Scavenging Assay

Using a 96-well plate, the samples were added, followed by the addition of the DPPH solution. For the control, only DPPH was added to the wells. This mixture was then incubated in the dark at room temperature for 30 min. The absorbance was measured at 517 nm using a microplate reader (Epoch Biotek, Agilent Technologies, Santa Clara, CA, USA) [39]. The percentage of DPPH radical scavenging was calculated using the following equation:

DPPH Radical Scavenging (%) = [ (A control − A sample)/A control] × 100.

#### 2.5.4. Copper Chelation

For this test, samples, 4 mM pirocatechol violet, and 50 µg/mL copper sulfate pentahydrate (in acetate buffer) were added to each well of the microplate. The copper ion chelation capacity was measured by reading the absorbance at 632 nm [40,41]. The percentage of copper chelation was calculated using the following equation:

Copper chelation (%) = [1 − (A sample/A blank)] × 100

### 2.6. Evaluation of Cytotoxic Activity on 3T3 Cell Line

#### 2.6.1. MTT Assay (3T3)

The EE and EA extracts were used to assess the effect of treatments on cell viability by reducing MTT to formazan. For this assay, the 3T3 cell line was cultured in 75 cm^2^ culture flasks containing DMEM culture medium supplemented with 10% fetal bovine serum (FBS). The cells were plated in a 96-well plate with 200 μL of DMEM at a density of 7 × 10^4^ cells/well and incubated for 24 h at 37 °C and 5% CO_2_. After this period, the medium was removed, and DMEM+FBS containing the treatments (EE and EA at concentrations of 50, 100, and 250 μg/mL) was added. Following a further 24 h incubation, the treatment medium was removed, and 5 mg/mL MTT reagent was added to each well. After 4 h of incubation, the insoluble formazan product was dissolved by adding 100 μL of absolute ethanol. The plates were gently shaken for 15 min. Subsequently, the absorbance was measured at 570 nm using a microplate reader [42]. The absorbance value obtained for the control (culture medium containing cells) was considered as representing 100% viability. The effects of the ethanolic and aqueous extracts on cell viability were then calculated as follows:

MTT reduction (%) = (abs. of sample 570 nm/abs. of control 570 nm) × 100

A statistically significant reduction of MTT was considered as cellular cytotoxicity.

#### 2.6.2. In Vitro Wound Healing Assay in 3T3 Cells

Initially, 3T3 cell line were plated at a density of 1 × 10^6^ cells/well in a 24-well plate using DMEM supplemented with 10% FBS and maintained at 37 °C in a 5% CO_2_ incubator. Upon reaching 80–90% confluence, the medium was aspirated, and the cells were washed with 1 mL of PBS (pH 7.4). A uniform “wound” was then created using a sterile 200 μL pipette tip. After creating the wound, the wells were washed again with PBS to remove detached cells and debris, and the liquid was aspirated. Samples of EE and EA at concentrations of 250 µg/mL were applied in triplicate. DMEM+FBS alone served as the negative control. Photographs of the wounds were taken using a Nikon Eclipse inverted microscope (Nikon Instruments Lnc., Melville, NY, USA) with a 40× objective at 0, 12, and 24 h post-application. Wound measurements were consistently taken from the same location within each well and analyzed using NIS-Elements AR software version 4.00.03 (Nikon Instruments Lnc., Melville, NY, USA, 2011). The results are expressed as the percentage of wound closure relative to the initial wound area [43].

Wound closure (%) = (initial area of wound − wound area after 0 h, 12 h or 24 h)/initial area of wound × 100%

#### 2.6.3. Copper Sulfate (CuSO_4_)-Induced Oxidative Stress Assay in 3T3 Cellular Model

The methodology for conducting this assay was adapted from [44] with modifications. Initially, a copper calibration curve was established to determine the optimal concentrations for the assay. The cells were seeded in 96-well plates at a density of 7 × 10^4^ cells/well in DMEM supplemented with 10% FBS and incubated at 37 °C and 5% CO_2_. After 24 h, serum deprivation was performed by replacing the medium with DMEM without FBS. Subsequently, this medium was aspirated, and DMEM+FBS containing various concentrations of copper (1–30 μM) along with 1 mM ascorbic acid was added to the wells for 45 min. Following exposure, the medium with the stressor was removed, and DMEM+FBS was replenished. After a 24 h recovery period, an MTT assay was performed. The concentration of copper selected for subsequent tests was 25 μM, based on its ability to reduce MTT by approximately 50%. In the copper-induced oxidative stress assay, under the same initial conditions, after serum deprivation, samples of EE and EA extracts at 100 μg/mL were added along with DMEM+FBS and 25 μM copper. After 45 min of exposure in the incubator, the medium was aspirated and replaced with fresh DMEM+FBS. Another 24 h of recovery was allowed before performing the MTT assay again. For controls, only DMEM+FBS was used for the negative control, and a combination of DMEM+FBS, 25 μM Copper Sulphate, and 1 mM Ascorbic Acid served as the positive control.

### 2.7. Evaluation of In Vivo Toxicity and Antioxidant Capacity

#### 2.7.1. Animal Model—*Tenebrio molitor*

*T. molitor* larvae were kept in black plastic boxes with dimensions of 30 × 45 × 15 cm at room temperature (25–29 °C). Their diet and substrate consisted of wheat bran, wheat flour, oat flakes, soy extract, and occasionally fruits [45].

#### 2.7.2. Toxicity Assay of Extracts in *T. molitor* Larvae

For the assay, initially, 10 larvae weighing between 100 and 150 mg were selected per treatment group. EE and EA extract samples were prepared at concentrations of 100 and 250 μg/mL with PBS. After separating the animals into petri dishes for their respective treatments, the animals were anesthetized on ice. Subsequently, 5 μL of EE and EA extracts per treated group was applied between the second and third segments on the ventral side of the hemocoel using a Hamilton syringe (Hamilton Company, Reno, NV, USA). The negative control was inoculated only with PBS. The experiment lasted for 10 days, counting the surviving larvae every 24 h. Larvae with no movement and that were melanized were considered dead. The assay was conducted in triplicate, based on [46].

#### 2.7.3. Copper Sulfate (CuSO_4_)-Induced Oxidative Stress Assay in *T. molitor*

For the assay, 10 *T. molitor* larvae weighing between 100 and 150 mg were selected per treated group (n = 30). The animals were placed in petri dishes and anesthetized on ice before the inoculations. Using a Hamilton syringe, 5 µL of CuSO_4_ at 25 µM was injected between the second and third segments on the ventral side to induce oxidative stress. With a 1 h interval, 5 µL of EE and EA extracts, both at 100 µg/mL, was injected. The negative control inoculations consisted only of PBS. For the positive control, the first inoculation contained 5 µL of CuSO_4_ at 25 µM, and after 1 h, the second inoculation was with 5 µL of PBS. The experiment lasted for 15 days, and survival was assessed every 24 h, as described above.

#### 2.7.4. Melanization Assay Following Copper Sulfate (CuSO_4_)-Induced Oxidative Stress in *T. molitor*

Melanization quantification was performed according to [47] with modifications. For each treatment group, five larvae were used across three biological replicates, totaling n = 15 larvae per treatment. Following 15 days of oxidative stress analysis, the larvae were anesthetized on ice and then stored at −20 °C for 2 h. Subsequently, using a scalpel blade, an incision was made in the first segment of the dorsal side of the head region to collect the internal contents of the larvae. The collected material was transferred to a 1.5 mL microtube. PBS was then added to the extracted content at a ratio of 1:100 (larvae/μL volume) and homogenized for 30 s on a shaker. The solution was subsequently centrifuged at 14.000 rpm (Eppendorf 5804R, Juelich, Germany) for 15 min at 4 °C. The supernatant was then used for melanization quantification, which involved measuring the absorbance at 405 nm using a spectrophotometer.

## 3. Results

### 3.1. Antioxidant Potential by Biochemistry Assay

The antioxidant effects of extracts from *G. decorticans* seeds were evaluated using four distinct tests, which assess the ability of biomolecules within the extracts to act as electron donors or acceptors.

**Figure 1:**
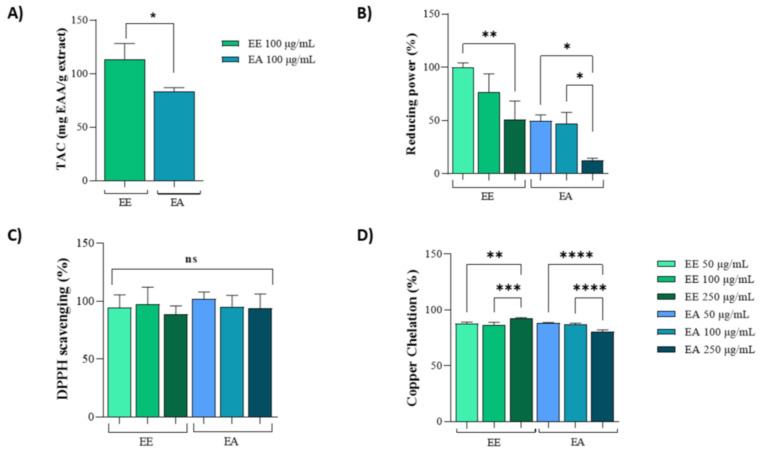
In vitro antioxidant capacity of extracts from *Geoffroea decorticans* seeds. Ethanolic Extract (EE); Aqueous Extract (EA). (**A**) Total Antioxidant Capacity (TAC). Results are expressed as mean ± standard deviation of ascorbic acid equivalents per gram of extract (mg AAE/mg extract). Effects between extracts were compared. (*) indicates statistical significance between EE and EA at a concentration of 100 µg/mL. (**B**) Reducing potential assay of EE and EA as electron donors. The *x*-axis corresponds to treatments with different EE and EA extracts at concentrations of 50, 100, and 250 µg/mL. The *y*-axis represents the percentage of activity based on the ascorbic acid standard curve. (* and **) denote significant differences between the evaluated concentrations of each extract. (**C**) DPPH free radical scavenging activity. The *x*-axis corresponds to treatments with different EE and EA extracts at concentrations of 50, 100, and 250 µg/mL. The *y*-axis represents the percentage of radical sequestration. (ns) indicates no statistically significant difference between the different evaluated concentrations of EE and EA. (**D**) Copper ion chelating activity. The *x*-axis corresponds to treatments with different EE and EA extracts at concentrations of 50, 100, and 250 µg/mL. The *y*-axis represents the percentage of copper chelation effect. (**, ***, and ****) denote significant differences between the evaluated concentrations. Assays were performed in triplicate for each extract, and statistical analysis was conducted using ANOVA and Tukey’s test (*p* ≤ 0.05).

The Total Antioxidant Capacity (TAC) assay was used to measure the general antioxidant potential. For this assay, a concentration of 100 µg/mL was selected for both the ethanolic (EE) and aqueous (EA) extracts. The results indicated that the antioxidant activity of EE was almost three times higher than that of the EA extract (Figure 1A). In the reducing power assay, both extracts exhibited similar activities at concentrations of 50 µg/mL and 100 µg/mL. However, EE consistently showed higher antioxidant activity compared to EA. Notably, the reducing power of both extracts decreased at a concentration of 250 µg/mL (Figure 1B).

Moreover, in the DPPH assay, both extracts exhibited identical activities at all three tested concentrations (50 µg/mL, 100 µg/mL, and 250 µg/mL), achieving 100% DPPH scavenging potential (Figure 1C). In the copper chelation assay, similar activities were observed between 50 µg/mL and 100 µg/mL for both the EE and EA extracts. However, at 250 µg/mL, there was a slight reduction in activity for the EA extract compared to the lower concentrations (Figure 1D).

### 3.2. Extract Composition

For the phenolic compounds, both extracts presented similar amounts (Table 1). On the other hand, the EE exhibited a significantly lower flavonoid content, about one-third less than the EA.

In addition, the identification of biomolecules using HPLC followed by GC-MS/SIM indicated the presence of phytol, vitexin, rutin, and α-tocopherol, as detailed in Table 2 and the Supplementary Tables and Figures (Table S1, Figures S1–S4). The concentrations of these compounds were similar in both extracts, as shown in Table 2.

### 3.3. In Vitro Assays Using NHI/3T3 Cell Line

**Figure 2:**
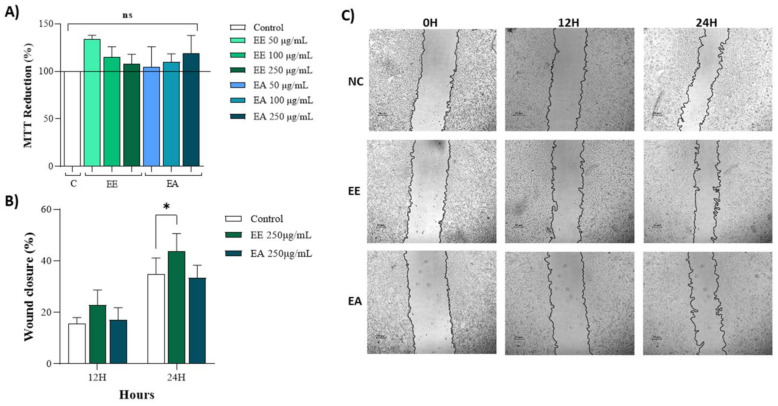
Cytotoxicity and wound healing of extracts from *Geoffroea decorticans* seeds. Ethanol Extract (EE); Aqueous Extract (EA); Control (C). (**A**) 3T3 cell capacity to reduce MTT. Control (C); Ethanol Extract (EE); Aqueous Extract (EA). Cells were exposed to treatments with EE and EA for 24 h. (ns) denotes no statistically significant difference between the different evaluated concentrations of EE and EA in relation to the Control and between each other. (**B**) Wound healing in 3T3 cells treated with EE and EA. (**C**) Representative photomicrographs of treatments and hours according to cell migration. Negative control (NC) contained only untreated cells. The concentration of the EE and EA used was (250 μg/mL). Measurements were taken at intervals of 0, 12, and 24 h. The areas were measured using NIS-Elements AR software. (*) indicates a significant difference between EE and the control at 24 h. Assays were performed in triplicate for each extract, and statistical analysis was conducted using ANOVA and Tukey’s test (*p* ≤ 0.05).

Both extracts, EE and EA, at concentrations of 50 µg/mL, 100 µg/mL, and 250 µg/mL exhibited no cytotoxicity to NIH/3T3 cells, as evidenced by nearly 100% MTT reduction (Figure 2A). The effects of these extracts on wound closure were also analyzed using plated cells to simulate an injury and assess the potential of these extracts to promote cell migration and enhance wound closure. At 12 h, there was a tendency for the extracts to stimulate cell migration, thus reducing the wound area (Figure 2B,C). However, by 24 h post-injury, no significant effects on cell migration or wound closure were observed (Figure 2B,C).

**Figure 3:**
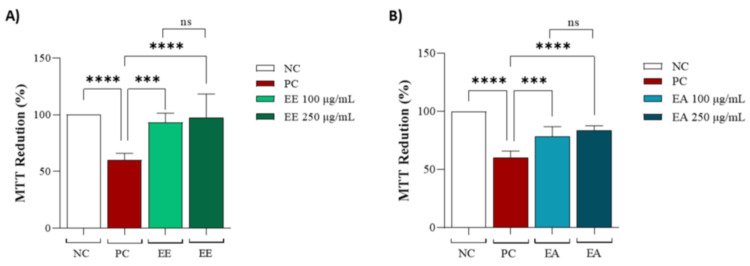
Protective capacity of *Geoffroea decorticans* seed extracts after copper sulphate treatment. (**A**) MTT reduction after copper sulphate-induced stress and treatment with (EE) at concentrations of (100 and 250 μg/mL). (**B**) MTT reduction after copper sulphate-induced stress and treatment with (EA) at concentrations of (100 and 250 μg/mL). Ethanol Extract (EE); Aqueous Extract (EA). Negative control (NC) contained only cells with culture medium; Positive control (PC) contained copper sulphate stressor (CuSO_4_) at 25 µM in the culture medium. (*** and ****) indicate significant differences between treatments and the positive control. (ns) denotes no statistically significant difference between EE and EA at their respective concentrations (100 and 250 μg/mL). Assays were performed in triplicate for each extract, and statistical analysis was conducted using ANOVA and Tukey’s test (*p* ≤ 0.05).

Both extracts, EE and EA, were evaluated in cell lines to determine whether they could protect against copper-induced oxidative stress. For the EE extract, no significant differences were observed between the different concentrations of 100 and 250 µg/mL, but it was effective in protecting the cell line against oxidative stress compared to the positive control (Figure 3A). The same conditions were observed for EA (Figure 3B).

### 3.4. In Vivo Assays Using Tenebrio molitor Animal Model

**Figure 4:**
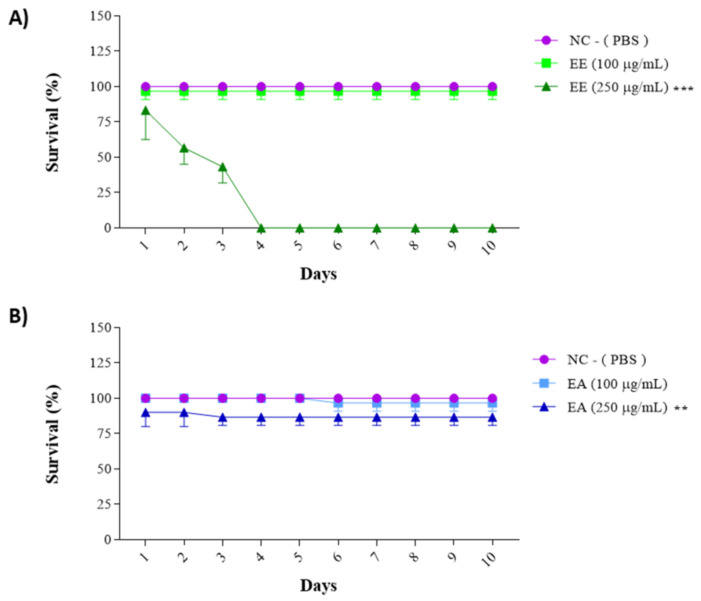
Survival assay with *Tenebrio molitor* assessing the toxicity of *G. decorticans* seed extracts. (**A**) Survival of *T. molitor* after inoculation with EE. (**B**) Survival of *T. molitor* after inoculation with EA. The concentrations evaluated in both extracts were (100 and 250 μg/mL). The survival assessment lasted for a period of 10 days. Ethanol Extract (EE); Aqueous Extract (EA); negative control (NC) inoculated only with PBS. Values were analyzed in triplicate and are expressed as mean ± standard deviation. The data were analyzed using the Dunnett test (** *p* < 0.001 and *** *p* < 0.0001) indicating significant differences between the extracts and the NC.

The previously obtained results for cell line treatments guided the choice of concentrations for this assay: 100 µg/mL and 250 µg/mL for both the EE and EA extracts from *G. decorticans*. The survival frequency was evaluated over 15 days. EE showed a 96.6% animal survival rate at a concentration of 100 μg/mL after 15 days (Figure 4). However, at a concentration of 250 μg/mL, EE exhibited statistically significant toxicity, with 100% mortality of all animals by the third day of the test (Figure 4A). In contrast, EA was not toxic at a concentration of 250 μg/mL. Similar to EE, EA at a concentration of 100 μg/mL was not toxic and also showed an average survival rate of 96.6%. At a concentration of 250 μg/mL, EA showed a survival rate of 86.6%, which was statistically different from the 13.4% survival rate in the control group. However, this rate was not considered toxic (Figure 4B). These results indicate that neither extract is toxic at concentrations of 100 μg/mL.

**Figure 5:**
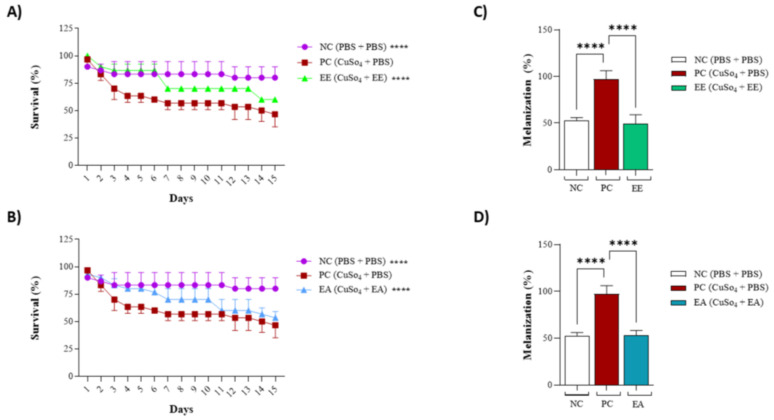
Protective effect of EE and EA extracts against copper sulfate-induced oxidative stress in *Tenebrio molitor*. (**A**) Survival of *T. molitor* after inoculation with EE following oxidative stress. (**B**) Survival of *T. molitor* after inoculation with EA following oxidative stress. The concentration evaluated for both extracts was (100 μg/mL). The survival assessment lasted for a period of 15 days. (**C**) Effect of EE on melanization of *T. molitor* after oxidative stress. (**D**) Effect of EA on melanization of *T. molitor* larvae after induction of oxidative stress. Ethanol Extract (EE); Aqueous Extract (EA); negative control (NC) inoculated only with phosphate-buffered saline (PBS); Positive control (PC) inoculated with copper sulfate (CuSO_4_) at 25 µM and PBS. Values were analyzed in triplicate and are expressed as mean ± standard deviation. The data were analyzed using the Dunnett test (**** *p* < 0.0001) indicating significant differences between the extracts and the PC.

As the extracts from *G. decorticans* were effective in protecting the 3T3 cell line against CuSO_4_-induced stress, the same activity was tested in an animal model. For both extracts, animal behavior mirrored the negative control for the first 4–5 days; subsequently, some deaths were observed, but at a lower rate than that of the positive control. Animals treated with EE or EA exhibited an intermediate mortality rate (Figure 5A,B), whereas deaths were observed starting from the third day in the positive control, maintaining a consistent 50% mortality rate (Figure 5A,B). Another aspect evaluated was the melanization frequency, associated with animal stress (Figure 5C,D). For the positive control, the melanization frequency was noted as 100%, while for the negative control, it was 50%, likely influenced by the animals’ reaction to PBS injections. Comparable to the negative control, the melanization frequency for treatments with EE or EA also registered at 50%, indicating that the extracts from *G. decorticans* effectively protected the animals against this oxidative stress (Figure 5C,D).

## 4. Discussion

Plants are a source of diverse biomolecules that can be used as complementary therapies for treating various diseases and for drug development [48,49]. Currently, medicinal plants are attracting attention in fields such as phytochemistry, pharmacology, and the health and economic sectors [50]. The data presented in this article show that two seed extracts, EE and EA, from *G. decorticans* (Chañar) were selected based on their traditional uses in folk medicine and their effectiveness in extracting phenolic compounds from seeds [51]. The effectiveness is further supported by the quantification of phenolic compounds in the EE and EA extracts, which was found to be higher than that reported by Reynoso et al. [22] for ethanolic, aqueous, and arrope (syrup) extracts of Chañar fruit and exceeded the results obtained by Somani et al. [23] for aqueous extracts.

The antioxidant activity is often associated with phenolic compounds and flavonoids due to their ability to act as ROS scavengers and free radical eliminators [52], providing nutritional advantages and protection against diseases generated by oxidative stress [50]. The presence of phenolic compounds was quantified in the ethanolic (EE) and aqueous (EA) extracts of Chañar seeds. Compared to previous studies, Reynoso et al. [22] reported values that were 74, 58, and 38 times lower in ethanolic, aqueous, and arrope extracts, respectively, from the fruit of *G. decorticans*. Similarly, Somani et al. [23] found values four times lower for the aqueous extract (52.27 ± 2.5 mg EAG/g extract) from the fruit of the same species. Additionally, extracts from the bran of cooked and fresh *G. spinosa* seeds showed lower values than those detected in the current study. These comparisons underscore the effectiveness of the extraction methodology utilized in this research.

The values reported in this study indicate that the EE and EA extracts from *G. decorticans* are rich in flavonoids, showing concentrations 7, 20, and 14 times higher than those obtained by Reynoso et al. [22] in ethanolic (EE), aqueous (EA), and arrope (EAr) extracts, respectively, prepared from the fruits of *G. decorticans*. Costamagna et al. [27] also identified flavonoids in ethanolic and aqueous extracts of *G. decorticans* fruit flour, albeit at levels lower than those reported here, specifically 90 ± 1 and 80 ± 0 mg QE/100 g. For *G. spinosa*, another plant of the same genus, the flavonoid levels found in cooked and fresh almond meal were close to those in the EE extract and lower for the EA, ranging from 10.42 to 10.97 mg/100 g. Overall, the findings suggest that the EE and EA extracts of Chañar are significant sources of flavonoids, which are crucial constituents of antioxidant activities and are dietary-derived, since the bioavailability of flavonoids accounts for two-thirds of the phenolics consumed [53].

The in vitro tests, Total Antioxidant Capacity (TAC) and Reducing Power, assess the ability of extracts to donate electrons and prevent oxidative damage through a similar mechanism. Both the EE and EA extracts demonstrated excellent electron-donating capacities, exhibiting antioxidant potential comparable to *Caesalpinia ferrea* extracts from the same *Fabaceae* family [54]. In the Reducing Power assay, the EE extract showed 100% antioxidant activity at the initial concentration of 50 µg/mL. Luna et al. [55] identified similar reducing capacities in the methanolic extract of *Ramorinoa girolae* (*Fabaceae*), albeit at higher concentrations (500 and 1000 µg/mL).

The chelating power of copper ions, essential in the oxidative stress propagation phase involving the reaction with hydrogen peroxide to generate hydroxyl radicals (OH-) [56,57], was effectively demonstrated by the EE and EA extracts of *G. decorticans* seeds across all tested concentrations. Jiménez-Aspee et al. [58] also observed strong metal ion chelation in phenolic-enriched methanolic extracts, capable of chelating copper and iron ions.

The reducing potential of flavonoids, largely attributed to the hydroxyl groups in their structures, has gained recognition for their role in chelating metal ions, as demonstrated with rutin found in the extracts [59,60]. Research suggests that flavonoids, with their antioxidant and copper-chelating activities, could serve as potential therapeutic agents for Alzheimer’s disease [61]. Furthermore, the chelating activity of *G. decorticans* EE and EA may be crucial in managing diseases related to metal accumulation, such as Wilson’s disease, which results from copper accumulation in metabolism [62].

Both extracts demonstrated excellent potential for scavenging DPPH radicals at all evaluated concentrations. Similar observations were made by Reynoso et al. [22] and Costamagna et al. [27], who noted that extracts from *Chañar* fruit and arrope also exhibited significant DPPH scavenging activity in vitro. Additionally, Jiménez-Aspee et al. [58] reported that the methanolic extract of *Chañar* fruits, enriched with phenolics, was effective in neutralizing DPPH radicals. Taken together, these results suggested that different extracts from *G. decorticans* fruits contain compounds that act to neutralize oxidizing agents.

The search for biomolecules that can be widely used for their pharmacological activities has focused on compounds that can be integrated into biochemical and pharmacological regulatory standards and are generated by plants. To assess the phytochemical composition of the EE and EA extracts of *G. decorticans* seeds, high-performance liquid chromatography (HPLC) followed by gas chromatography-mass spectrometry in selective ion monitoring mode (GC-MS/SIM) was carried out, showing the presence of phytol, vitexin, rutin, and α-tocopherol.

Phytol, a diterpene, is widely recognized for its antimicrobial, anticonvulsant, antinociceptive, anti-inflammatory, and antioxidant activities [63,64,65,66] and is used in the cosmetics and food industries [67]. The antioxidant activity of phytol is attributed to its ability to scavenge free radicals, facilitated by the hydrogen in its alcohol group and the stabilization offered by its double bonds in the resonance structure of the radicals [68]. Additionally, studies involving B16F10 murine melanoma cells indicate that phytol may reduce hyperpigmentation by inhibiting melanogenesis. It achieves this by suppressing the expression of tyrosinase and TRP1 protein, with tyrosinase being a critical rate-limiting enzyme required for melanogenesis [69]. Shukla et al. [70] identified phytol as a hepatoprotective compound, considering its beneficial effects on the human body, and suggesting its potential as a new drug and treatment for liver dysfunction. Furthermore, the hepatoprotective mode of action of phytol may be related to its antioxidant activity, as well as its synergistic effects. This action was confirmed through pre-clinical evaluation of its hepatoprotective activity using albino Wistar rats, where a reduction in the elevated levels of SGPT, SGOT, ALP, TG, cholesterol, and bilirubin was observed. Additionally, phytol reduced the levels of TP, SOD, CAT, and GSH in diseased animals. Given its broad spectrum of action, phytol is considered safe for use as a therapeutic option, both orally and intravenously [71]. Sakthivel et al. [72] also noted phytol’s potential as a regulator of antioxidant enzymes, as well as its ability to protect cells against B(a)P-induced carcinogenesis, without displaying any adverse toxic effects in animals.

Known as a key member of the vitamin E family, α-tocopherol is characterized by its fat solubility and potent antioxidant properties. It primarily acts by neutralizing hydroxyl radicals (OH•) and singlet oxygen (^1^O_2_) and reducing lipid peroxidation [73]. Impressively, one molecule of *α*-tocopherol can deactivate up to 120 singlet oxygen species before being consumed [74]. This compound is vital for plants, animals, and humans, who obtain these non-enzymatic antioxidant compounds through their diet [75]. Tocopherols are generally extracted from the fruits and seeds of medicinal plants using ethanolic extracts [76], as demonstrated in the EE and EA extracts of *Geoffroea decorticans* seeds in this study. Furthermore, Cittadini et al. [77] identified the presence of *α-tocopherol* in hexane extracts of *G. decorticans* seeds. Consequently, the consumption of *G. decorticans*, whether fresh, as a tincture (ethanolic extract), or an infusion (aqueous extract), provides an excellent source of antioxidants, underscoring the significance of these biomolecules as identified in this work and corroborated by the scientific literature. McCluskey et al. [78] observed that the administration of α-tocopherol significantly improved cell viability by decreasing SOD and CAT activities to normal levels in porcine ovarian granulosa cells treated with cholestanetriol, demonstrating its therapeutic antioxidant potential in vitro. Trials conducted on catfish (*Clarias gariepinus*) exposed to different concentrations of deltamethrin showed an increase in MDA levels in the liver, kidneys, and gills. These effects are linked to deltamethrin’s ability to generate free radicals, reduce CAT activity, decrease total protein and albumin levels, among other complications, suggesting that deltamethrin induces liver dysfunction [79]. However, the administration of α-tocopherol exhibited a protective effect in catfish, which may be important for shielding various tissues from oxidative damage caused by deltamethrin. This protective effect might also be relevant to its prophylactic and therapeutic potential against environmental pollutants [79]. Studies by [80] highlight the protective effect of α-tocopherol against damage induced by hexavalent chromium (Cr VI) in the liver and kidneys by inhibiting lipid peroxidation through its antioxidant activity. Additionally, research has shown that dietary supplementation with α-tocopherol reduces susceptibility to lipid peroxidation in rat tissues, as well as Fe/ascorbate-induced peroxidation of rat liver microsomes, emphasizing the importance of adequate vitamin supplementation for the body under stress [81]. The other molecule identified in both EE and EA was vitexin, an 8-C glycoside derived from apigenin commonly found in foods and many medicinal plants [82]. The presence of vitexin has also been identified in mung bean seeds (*Vigna radiata*), a species from the same family as Chañar (Fabaceae) [83]. This biomolecule is known for its diverse pharmacological activities, including anti-inflammatory, antioxidant, antiviral, neuroprotective, and antinociceptive effects [84,85,86]. Its antioxidant activity is associated with its ability to donate electrons, acting as an ROS eliminator [85]. Vitexin is already recognized as a dietary polyphenol with excellent pharmacological activities. The exposure of animals to the heavy metal cadmium led to an increase in lipid peroxidation in cells, compromising the plasma membrane of hepatocytes and causing the disordered release of liver function enzymes (ALT, AST, and ALP) into the blood, along with total bilirubin, indicating liver dysfunction [87]. However, treatment with vitexin reduced the levels of these liver markers, demonstrating vitexin’s ability to restore the structural integrity of the hepatocyte plasma membrane and reduce ROS levels, lipid peroxidation, and total bilirubin [88]. In line with in vitro studies, vitexin also alleviated renal fibrosis, damage, and ferroptosis in rats with diabetic nephropathy. Additionally, trials using vitexin as a pre-treatment showed a reduction in fibrosis (collagen type I Col I, TGF-β1), as well as a decrease in ferroptosis, ROS, Fe^2+^, and MDA levels, with an increase in GSH levels in rats with diabetic nephropathy [89]. The protective and healing potential of vitexin was further observed in studies with male albino rats, where vitexin was shown to mitigate Cisplatin-induced nephrotoxicity, reaffirming its ability to restore the activity of antioxidant enzymes by scavenging radicals [90].

Another compound identified in the EE and EA extracts, belonging to the flavonoids group, was rutin. This substance is often used as a standard to assess the antioxidant activity of crude extracts and is recognized for its diverse pharmacological properties, including cardioprotective, anti-inflammatory, anticancer, antiallergic, antidiabetic, and antioxidant effects [91]. Arjumand et al. [92] evaluated the protective effects of rutin against cisplatin-induced nephrotoxicity in rats and observed that rutin mitigated cisplatin-induced lipid peroxidation, xanthine oxidase (XO) activity, and GSH depletion. Studies using LLCPK1 renal tubular epithelial cells also evaluated the effect of rutin against vancomycin-induced stress, mitochondrial dysfunction, and apoptosis, showing that rutin decreased oxidative stress, thereby providing a protective effect against nephrotoxicity [93]. Additionally, rutin administration was able to reverse kidney damage caused by the stressor LPS in C57BL/6 mice [94]. Due to its broad range of action, rutin exhibits various pharmacological effects, including protective effects on the liver and kidneys against synthetic stressors and toxins with different mechanisms of action. The beneficial effects of rutin are directly related to its antioxidant capacity, promoting increased activity of enzymes such as SOD, GST, GGT, CAT, and GPx GR [93]. Consequently, rutin can be considered a potential nutraceutical for managing acute organ dysfunctions caused by inflammatory and oxidative damage [95].

The presence of rutin, along with other compounds in the EE and EA extracts of *G. decorticans* seeds, contributes to their potent antioxidant capabilities. These extracts contain a range of bioactive substances from terpenes to polyphenolics, which are known to act as antioxidants either individually, in synergy, or in combination with vitamins. Together, they protect human body tissues from the harmful effects of oxidative stress and help mitigate diseases generated by this metabolic condition [96].

The safety of the EE and EA extracts was assessed through cytotoxicity evaluations using the 3T3 mouse embryonic fibroblast cell line (ATCC CRL-1658). This cell line was chosen considering its relevance in processes such as inflammation, cell proliferation, and migration, which are crucial for wound healing and typically involve fibroblasts. The results demonstrated that the extracts, at various concentrations, did not exhibit cytotoxicity or compromise the metabolic activity of the 3T3 fibroblast cell line, thus confirming their safety for further trials. Additionally, the non-cytotoxic nature of these extracts was supported by similar findings from an MTT assay conducted on extracts from the leaves and roots of *Christia vespertilionis*, a plant from the same Fabaceae family as *G. decorticans* [97].

To assess the impact of the EE and EA extracts on the cell healing process, given their demonstrated lack of cytotoxicity, antioxidant capacity, and anti-inflammatory activity cited by other research groups [58,98], this study found that differences of about 7 to 9% from the negative control at 12 and 24 h were not statistically significant for either extract. There is a lack of data in the literature regarding the action of *G. decorticans* on cell proliferation in fibroblasts or other cell types involved in the healing process. Although the extracts did not induce cell migration or affect cell proliferation compared to the negative control, these findings confirm the non-cytotoxic nature of the extracts, as they do not interfere with cell growth. However, other members of the Fabaceae family, such as *Caesalpinia sappan* and *Astragalus propinquus*, have been shown to promote the proliferation and migration of dermal fibroblasts, thereby aiding in the healing processes of cutaneous and diabetic wounds [99,100]. Moreover, some medicinal plants are recognized as sources of healing, acting as therapeutic agents for wound treatment and skincare [101,102]. For instance, *Aloe vera* is utilized for wound healing due to its bioactive molecules, such as phytol [103].

Oxidative stress can arise from exposure to various agents such as hydrogen peroxide, UV radiation, and heavy metals, including copper [104]. Copper is an essential trace element for critical cellular functions within the body [105]. Nonetheless, excessive copper can lead to toxic effects, and prolonged exposure is linked to the progression of oxidative stress. This includes increased levels of reactive oxygen species (ROS), superoxide dismutase (SOD), catalase (CAT), lipid peroxidation (LPO), and hydroxyl radicals (OH•), alongside a decrease in endogenous antioxidant enzymes such as copper-zinc superoxide dismutase (CuZn-SOD). Such imbalances can damage mitochondrial dynamics, compromise redox homeostasis, and induce apoptosis across various cell types [106,107,108,109]. Copper accumulation can lead to diseases such as liver disorders, Wilson’s disease, neurodegenerative conditions (Alzheimer’s and Parkinson’s), cardiovascular issues, and cancer, among others [105,110,111].

Considering the in vitro copper-chelating potential of the EE and EA extracts, this activity was further evaluated in the 3T3 cell line under oxidative stress induced by copper sulfate. In this assay, both extracts demonstrated protective activity against oxidative stress, likely due to the bioactive compounds identified during phytochemical characterization. These biomolecules may act to eliminate or neutralize free radicals generated by copper sulfate, including those produced by the Fenton and Haber–Weiss reactions, which form hydroxyl radicals (OH•). Copper chelation is effective during both the initiation and propagation stages of free radical production [44]. The bioactive compounds in the EE and EA extracts, such as tocopherols, carotenoids, and phenolics, might function either individually or synergistically to provide antioxidant protection against copper-induced stress [112,113,114]. Presa et al. [44] also employed copper sulfate as an oxidative stress inducer in 3T3 fibroblast cells, observing protection from induced stress by sulphated polysaccharides. These findings suggest that the EE and EA extracts from *G. decorticans* could potentially be utilized to manage or prevent conditions associated with oxidative stress and excessive copper in the body, including rheumatic and neurodegenerative disorders, liver problems, Wilson’s disease, cardiovascular issues, and cancer [62,105,111].

The use of cell lines is an excellent model for assessing cytotoxicity and the effects of extracts. Nevertheless, employing animal models is crucial to understand how extracts may act across different cells, tissues, and signaling pathways and to evaluate their safety concerning the toxicity of certain compounds after body metabolization [115]. An invertebrate model was chosen for its short life cycle and ease of maintenance [116,117]. The *T. molitor* model, with its manageable size, allows for precise inoculation of extracts directly into the animal, making it ideal for assessing toxicity, conducting pre-clinical research, and evaluating the virulence of fungi and bacteria. Furthermore, this invertebrate has been utilized to assess the toxicity of various medicinal compounds. For instance, Brai et al. [115] found that drugs such as nimesulide, paracetamol, amoxicillin, and cloxacillin were toxic to this model, underscoring its utility. Additionally, *T. molitor* possesses a defense system with well-documented signaling pathways, including enzymatic cascades such as phenoloxidase-PO, which facilitates a comprehensive assessment of antioxidant activity [46,115,118].

The toxicity evaluation of the EE and EA extracts from *G. decorticans* indicated that the optimal concentration for use was 100 µg/mL. Similarly, trials using hydroethanolic extracts and infusions of the *Talisia esculenta* plant, tested at the same concentration on the *T. molitor* model, also demonstrated no toxicity to the insect [119]. Conversely, the toxicity observed at a concentration of 250 µg/mL for the EE extract of *G. decorticans* might be linked to high amounts of phenols, as suggested by [120].

Given that heavy metals such as copper sulfate (CuSO_4_) can induce oxidative stress in animals [121,122], the *T. molitor* model was utilized to assess the protective effects of the EE and EA extracts. These effects were previously observed in the 3T3 cell line. Silva et al. [46] also observed that this invertebrate model can be used for antioxidant assays. Observations indicated that the larvae treated with these extracts exhibited higher survival rates compared to the untreated larvae (negative control, inoculated only with PBS). This protection may be attributed to the presence of biomolecules in the extracts, which minimize alterations in the balance of pro- and antioxidants induced by free radical production [122]. Similar protective effects have been reported in other studies. For instance, extracts from *Buchenavia tetraphylla* leaves protected *T. molitor* larvae against oxidative stress induced by inactivated *Escherichia coli* [46], and extracts from *Talissia esculenta* shielded *T. molitor* larvae against oxidative stress induced by copper sulfate [118].

It has been verified that *T. molitor*, when subjected to mechanical injuries, parasites, or pathogens, can activate an antioxidant cascade. This involves the activation of various enzymes, subsequently triggering the insect’s innate immunity [120]. Due to the absence of immunoglobulins in their metabolism, insects have evolved to utilize the melanin biosynthetic pathway as a defense mechanism against infections, thereby enhancing their immune response [123].

Key enzymes such as tyrosinase and phenol oxidase (PO) are integral to the immune response and the melanization process. Melanin production not only restricts oxygen and nutrient availability to pathogens, thereby affecting their survival, but the melanization process also generates reactive oxygen species (ROS) and reactive nitrogen species (RNS). These potentially harmful molecules need to be neutralized to prevent cellular damage. To counteract these effects, insects employ detoxifying enzymes and endogenous antioxidants, including superoxide dismutase, peroxidases, catalases, tyrosinase, acetylcholinesterase, carboxylesterase, and glutathione S-transferase. Exogenous antioxidants in their diet can further reinforce these defenses [46].

Despite the benefits of releasing certain toxic substances to combat invasive organisms and injuries, these substances can also harm the insects, potentially reducing their longevity. In studies involving the Chañar extracts, it was observed that these extracts reduced the melanization content by approximately 45% in treated animals compared to those treated only with copper sulfate. Additionally, phytol, a compound identified during phytochemical characterization, may contribute to this reduction by inhibiting tyrosinase activity and the expression of proteins involved in melanogenesis [69].

## 5. Conclusions

This study demonstrates the antioxidant potential and protective effects against oxidative stress induced by copper sulfate. The ethanolic (EE) and aqueous (EA) extracts derived from the seeds of *G. decorticans* exhibited remarkable activity across various methodologies, including biochemical assays, in vitro studies, and experiments using the 3T3 cell line and *T. molitor* as an animal model. In addition, the results provide robust evidence supporting the potential use of these seeds or extracts as a supplementary dietary source. This recommendation is bolstered by the observed antioxidant activities and the phytochemical composition identified, which includes compounds such as phytol, vitexin, rutin, and α-tocopherol (vitamin E). These bioactive compounds are capable of acting individually or synergistically, contributing to the activities evaluated in this study.

## Figures and Tables

**Table 1:** Determination of phytochemical components of *Geoffroea decorticans* seeds.

Compounds	*G. decorticans* Extracts	
	EE	EA
Total Phenolic Compounds (mg EAG/g Extract)	238 ± 5 a	211 ± 3 b
Total Flavonoids (mg EQ/g Extract)	13.7 ± 2 a	35.4 ± 3 b

Ethanolic extract (EE); Aqueous extract (EA); Gallic acid milligram equivalents/gram extract (mg EAG/g Extract); Quercetin milligram equivalents/gram extract (mg EQ/g Extract). The data are expressed as the mean, standard deviation (±) and were analyzed by one-way statistical analysis of variance (ANOVA), followed by Tukey’s test. Different letters (a, b) mean differences between the extracts for the different tests carried out.

**Table 2:** Quantification of phenolic compounds by HPLC-(GC-MS/SIM) of the EE and EA of *Geoffroea decorticans* seeds.

Compounds	*G. decorticans* Extracts	
	EE	EA
Phytol	10.94 ± 0.07 ng·mL^−1^ a	2.34 ± 0.31 ng·mL^−1^ b
Vitexin	18.17 ± 0.08 ng·mL^−1^ a	1.56 ± 0.05 ng·mL^−1^ b
Rutin	28.13 ± 0.23 ng·mL^−1^ a	25.07 ± 0.16 ng·mL^−1^ b
α-Tocopherol	5.51 ± 0.36 ng·mL^−1^ a	4.88 ± 0.09 ng·mL^−1^ b
Amonafide	nd	nd
Caffeic acid	nd	nd
Isovitexin	nd	nd
Epifriedelanol	nd	nd
Leucocyanidin	nd	nd

*Geoffroea decorticans* (G.D.); Ethanolic extract (EE); Aqueous extract (EA); Not detected (nd). The data are expressed as the mean, standard deviation (±) and were analyzed by one-way statistical analysis of variance (ANOVA), followed by Fisher’s difference test (LSD). All treatments were arranged in a completely randomized design with three replications. The data were analyzed using SigmaPlot 11.0 software (Systat Software, Richmond, CA, USA). In all cases, differences were considered significant when (*p* < 0.05). Averages followed by identical letters do not differ between treatments (a, b).

## Data Availability

The original contributions presented in the study are included in the article/Supplementary Materials, further inquiries can be directed to the corresponding authors.

## Supplementary Materials

The following supporting information can be downloaded at: https://www.mdpi.com/article/10.3390/nu16172813/s1, Supplementary Table S1: Detection of biocompounds present in EE and EA extracts in *Geoffroea decorticans* seeds by GC-MS/SIM. Supplementary Figure S1: Gallic acid for total phenolic compounds. Supplementary Figure S2: Phytol Spectra identification In (A) is represents the Sample spectrum. In (B) represents the Standard spectrum and in (C) it is shown the ions confirmation and their fragmentations, in red is the sample and in black the standard. Supplementary Figure S3: Vitexin Spectra identification In (A) is represents the Sample spectrum. In (B) represents the Standard spectrum and in (C) it is shown the ions confirmation and their fragmentations, in red is the sample and in black the standard. Supplementary Figure S4: Rutin Spectra identification In (A) is represents the Sample spectrum. In (B) represents the Standard spectrum and in (C) it is shown the ions confirmation and their fragmentations, in red is the sample and in black the standard. Supplementary Figure S5: Alpha-tocopherol Spectra identification In (A) is represents the Sample spectrum. In (B) represents the Standard spectrum and in (C) it is shown the ions confirmation and their fragmentations, in red is the sample and in black the standard.
