# Supplementary material for: Prospecting of the Antioxidant Activity from Extracts Obtained from Chañar (Geoffroea decorticans) Seeds Evaluated In Vitro and In Vivo Using the Tenebrio molitor Model
Source: Nutrients. 2024 Aug 23;16(17):2813. doi: 10.3390/nu16172813 (PMC11396818; doi:10.3390/nu16172813)
Supplement: Supplementary file 1 [file nutrients-16-02813-s001.zip › nutrients-3118712-supplementary.pdf]

Supplementary Table S1: Detection of biocompounds present in EE and EA extracts in *Geoffroea decorticans* seeds by GC-MS / SIM.

Substance	RT	Sample/standard	m/z (%, relative intensity of base peak)		
Phytol	21.60	Sample	70 (100)	54 (7)	46 (19)
		Standard	71 (100)	55 (9)	45 (21)
Vitexin	18.20	Sample	430 (100)	402 (20)	392 (8)
		Standard	431 (100)	404 (10)	390 (7)
Rutin	20.70	Sample	297 (100)	282 (66)	288 (63)
		Standard	300 (100)	283 (71)	290 (68)
α-Tocopherol	24.50	Sample	162 (100)	212 (20)	185 (8)
		Standard	165 (100)	215 (20)	186 (6)

Supplementary Figure S1 – Gallic acid for total phenolic compounds

Gallic acid curve (mg/mL)	
Conc. de A G	Abs.
0,1	0,013917
0,25	0,029
0,5	0,0555
1	0,11525
2	0,22075
3	0,4685
4	0,53525
5	0,77725

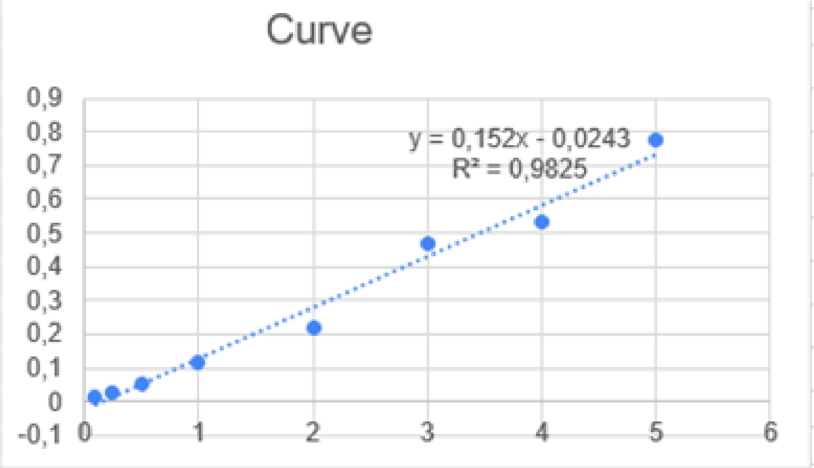

Phytol Spectra

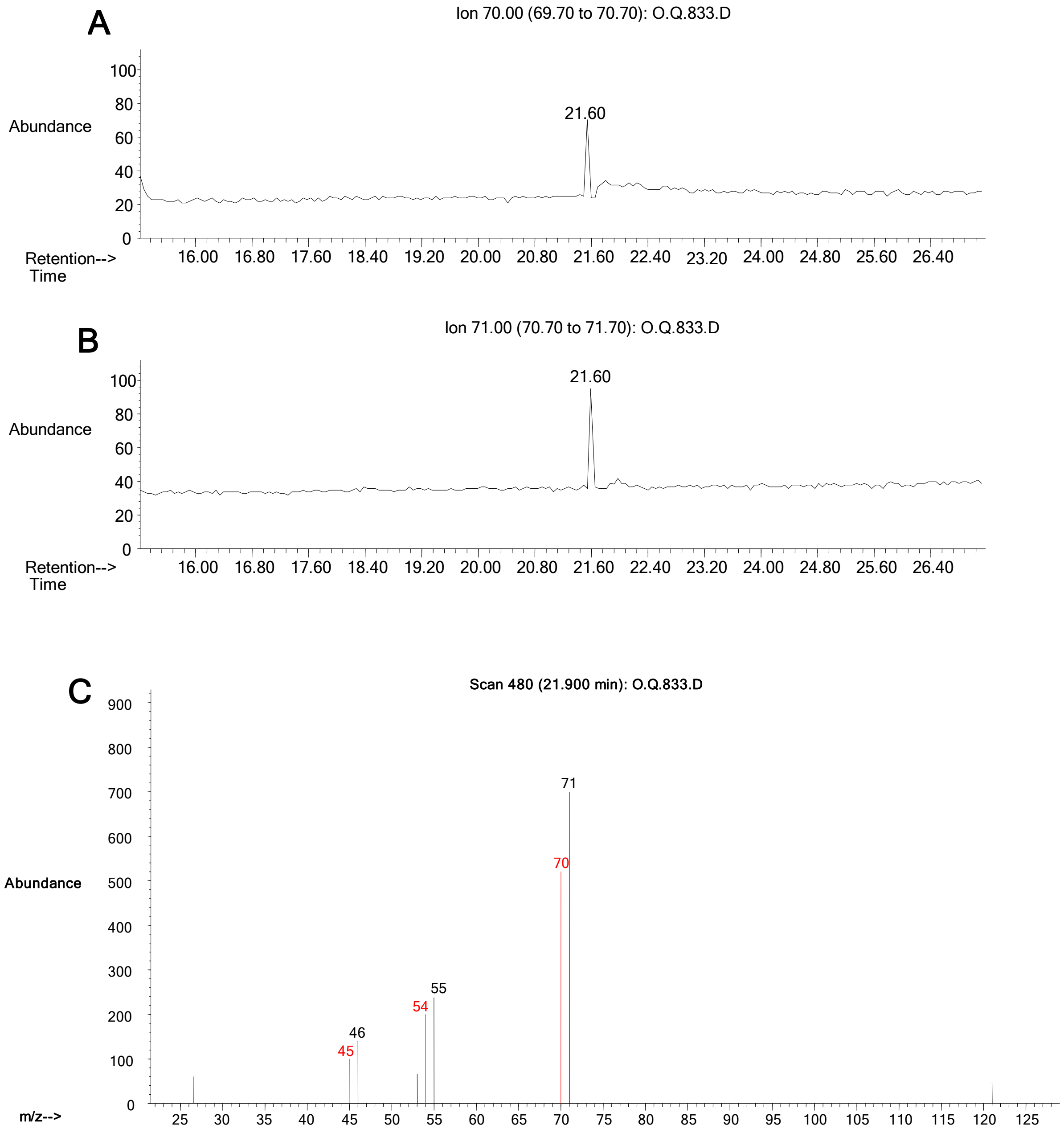

Vitexin Spectra

A

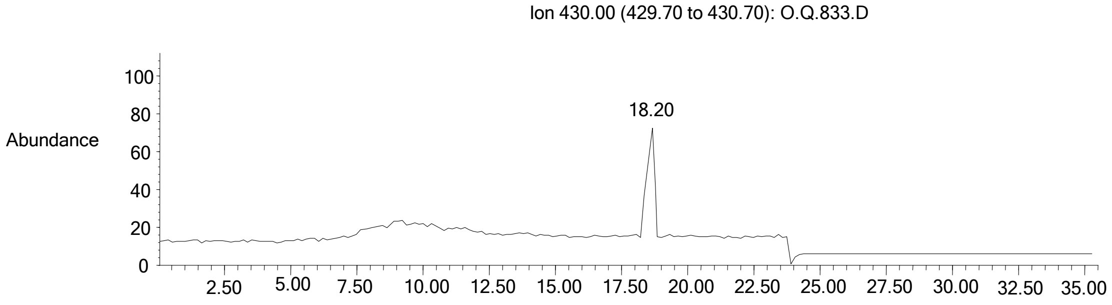

B

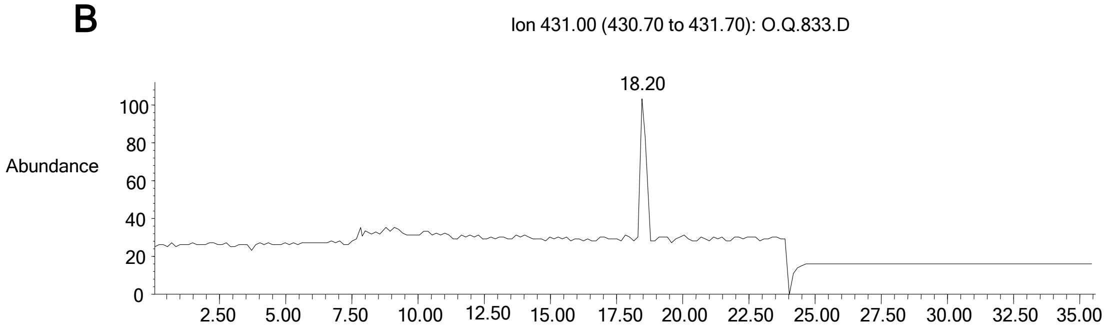

C

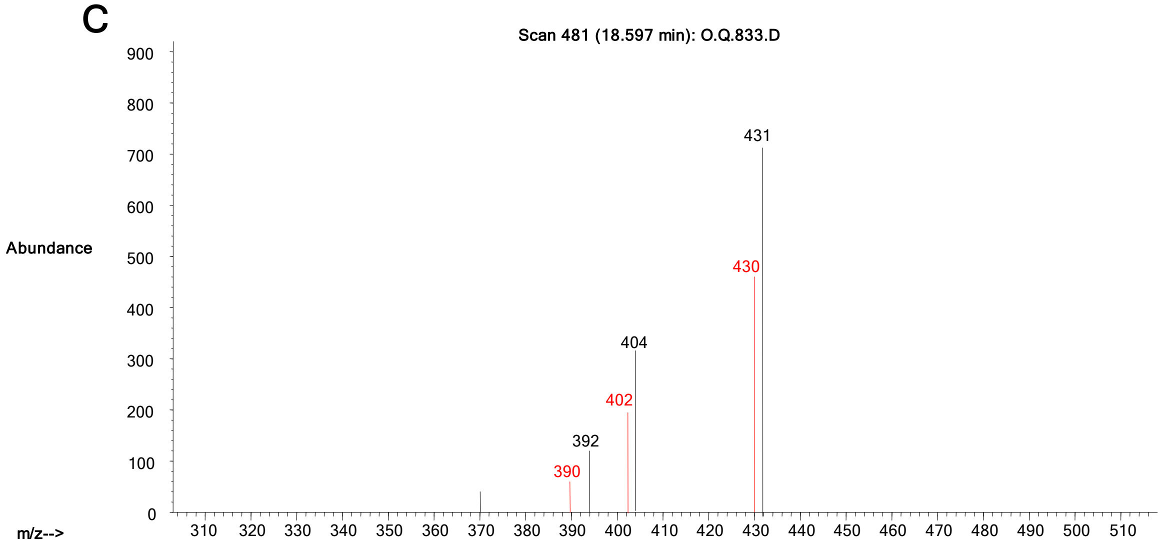

Rutin Spectra

A

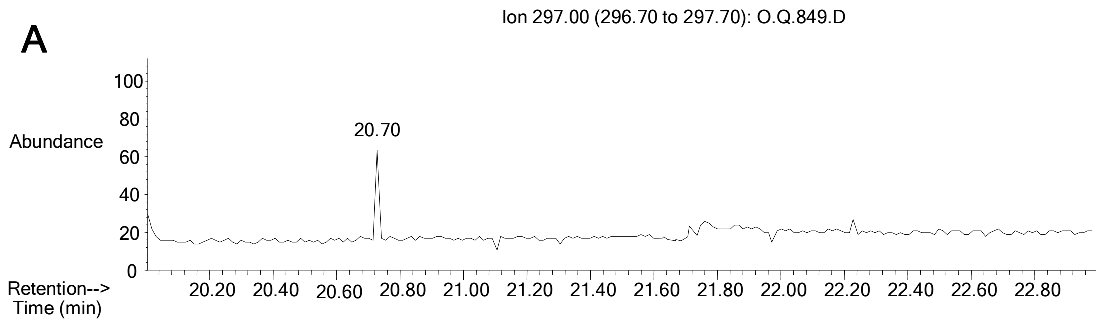

B

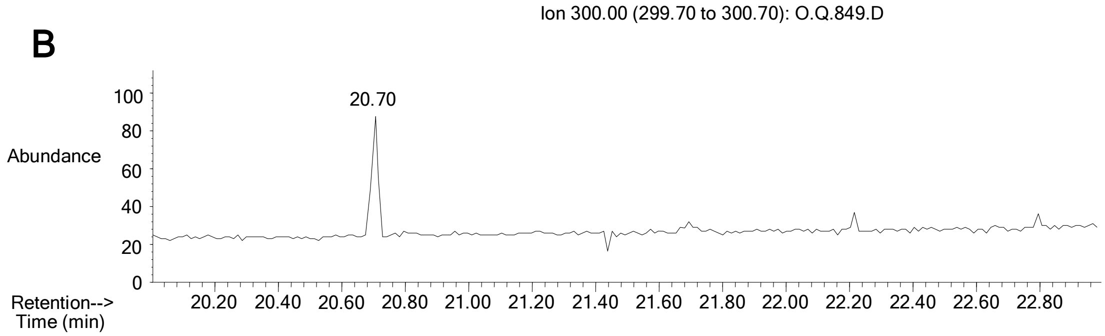

C

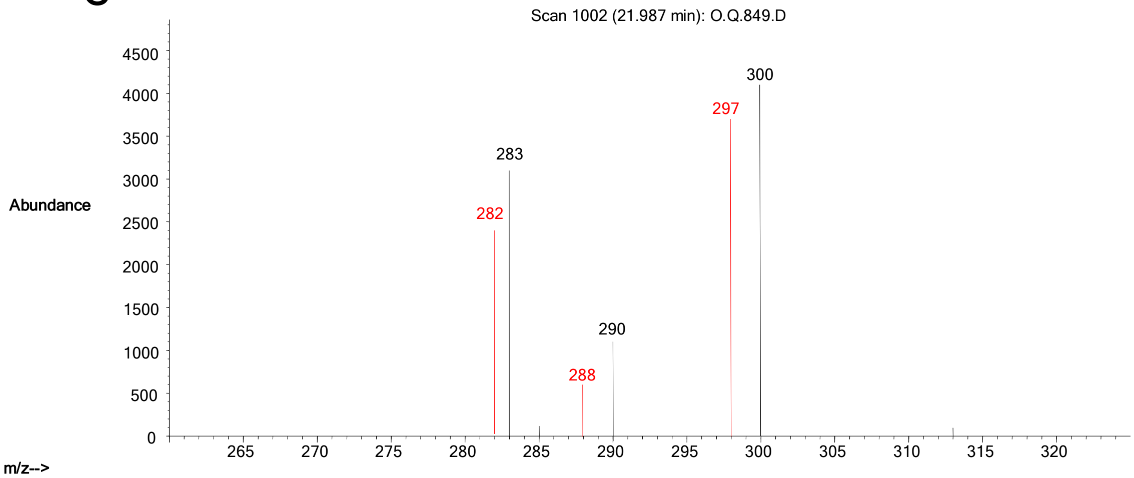

α-Tocopherol Spectra

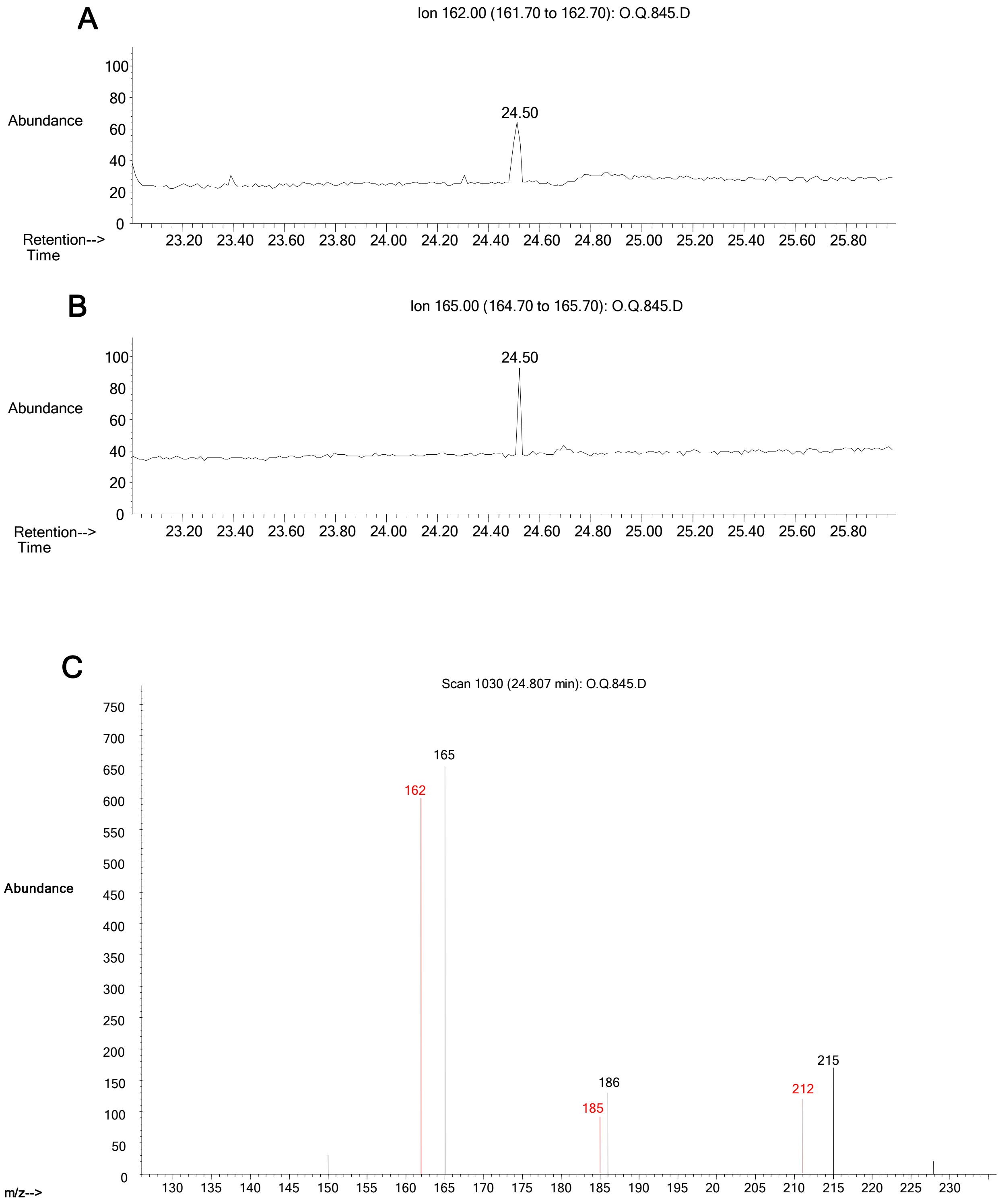

Supplementary Figure Legends

Supplementary Figure S2 – Phytol Spectra identification In (A) is represents the Sample spectrum. In (B) represents the Standard spectrum and in (C) it is shown the ions confirmation and their fragmentations, in red is the sample and in black the standard.

Supplementary Figure S3 – Vitexin Spectra identification In (A) is represents the Sample spectrum. In (B) represents the Standard spectrum and in (C) it is shown the ions confirmation and their fragmentations, in red is the sample and in black the standard.

Supplementary Figure S4 – Rutin Spectra identification In (A) is represents the Sample spectrum. In (B) represents the Standard spectrum and in (C) it is shown the ions confirmation and their fragmentations, in red is the sample and in black the standard.

Supplementary Figure S5 – Alpha-tocopherol Spectra identification In (A) is represents the Sample spectrum. In (B) represents the Standard spectrum and in (C) it is shown the ions confirmation and their fragmentations, in red is the sample and in black the standard.
